# Genetics, Cognition, and Neurobiology of Schizotypal Personality: A Review of the Overlap with Schizophrenia

**DOI:** 10.3389/fpsyt.2014.00018

**Published:** 2014-02-21

**Authors:** Ulrich Ettinger, Inga Meyhöfer, Maria Steffens, Michael Wagner, Nikolaos Koutsouleris

**Affiliations:** ^1^Department of Psychology, University of Bonn, Bonn, Germany; ^2^Department of Psychiatry and Psychotherapy, University of Bonn, Bonn, Germany; ^3^Department of Psychiatry and Psychotherapy, University of Munich, Munich, Germany

**Keywords:** schizophrenia, schizotypy, personality, phenotype, spectrum, genetics, neuroimaging, cognition

## Abstract

Schizotypy refers to a set of temporally stable traits that are observed in the general population and that resemble the signs and symptoms of schizophrenia. Here, we review evidence from studies on genetics, cognition, perception, motor and oculomotor control, brain structure, brain function, and psychopharmacology in schizotypy. We specifically focused on identifying areas of overlap between schizotypy and schizophrenia. Evidence was corroborated that significant overlap exists between the two, covering the behavioral brain structural and functional as well molecular levels. In particular, several studies showed that individuals with high levels of schizotypal traits exhibit alterations in neurocognitive task performance and underlying brain function similar to the deficits seen in patients with schizophrenia. Studies of brain structure have shown both volume reductions and increase in schizotypy, pointing to schizophrenia-like deficits as well as possible protective or compensatory mechanisms. Experimental pharmacological studies have shown that high levels of schizotypy are associated with (i) enhanced dopaminergic response in striatum following administration of amphetamine and (ii) improvement of cognitive performance following administration of antipsychotic compounds. Together, this body of work suggests that schizotypy shows overlap with schizophrenia across multiple behavioral and neurobiological domains, suggesting that the study of schizotypal traits may be useful in improving our understanding of the etiology of schizophrenia.

## Introduction

Schizophrenia is a serious psychiatric condition with unknown etiology characterized by positive symptoms (such as hallucinations and delusions), negative symptoms (such as avolition and psychomotor poverty), and thought disorder as well as cognitive deficits. The illness has adverse consequences not only for the sufferers but also their relatives and society at large (1). Patients with schizophrenia are at significantly increased risk of suicide and frequently suffer socio-economic disadvantages. The costs to society include direct costs, such as treatment, as well as indirect costs, such as loss of manpower at work due to the illness or due to caring by relatives (2, 3). Current pharmacological treatments of schizophrenia are successful at reducing psychotic symptoms but do not provide a cure (4), especially given that neurocognitive deficits and negative symptoms appear less amenable to treatment than psychotic phases of the disorder.

An important step in advancing etiological research into this condition was the realization that schizophrenia is not, despite its clinically important and reliable categorical diagnosis according to the International Classification of Disease (ICD) of the World Health Organization or the Diagnostic and Statistical Manual (DSM) of the American Psychiatric Association, a binary phenotype (present, absent) with sudden disease onset. Instead, there is substantial agreement amongst clinicians and researchers that the systematic investigation of intra- and inter-individual continua plays an important role in improving our understanding of the etiology of the disorder (5–7).

There are at least three different types of continua to be distinguished in this context: first, retrospective and longitudinal studies have provided evidence for a temporal continuum. For example, early motor and cognitive deficits as well as individual psychotic symptoms are observed in childhood and youth of those who later develop schizophrenia (8, 9). These signs and symptoms dramatically increase in the prodrome, immediately before illness onset (10).

Second, clinical studies of patients with schizophrenia have shown that even within the clinically meaningful diagnostic borders of schizophrenia, significant inter-individual differences exist both in the kind and severity of symptoms as well as in the longitudinal course of the illness. In an influential study, Strauss (11) observed in a sample of patients that symptoms such as hallucinations or delusions could often not be classified as unambiguously present or absent. This observation was interpreted as a contradiction to the assumption of a binary illness phenotype [(11); p. 585]: “Hallucinations and delusions are key symptoms in the conceptualization and diagnosis of schizophrenia. In the past, their definition as discrete symptoms has been used to substantiate the supposedly discrete nature of this disorder. Recognizing these symptoms as points on continua implies that schizophrenia too might be more adequately described as a point or a series of points on a functional continuum.”

Third, and of most relevance to this article, a large and continuously growing body of evidence suggests that certain aspects of the phenomenology of schizophrenia are also traceable in the general population, beyond the diagnostic borders of the current nosological systems ICD and DSM (12). One of the first empirical investigations of this issue (13) observed in a sample of approximately 17,000 adults in Victorian England that about 8% of males and 12% of females reported at least one hallucination in their lifetime [see also Ref. (14)]. More recent systematic investigations by van Os and colleagues similarly document the existence of individual psychotic symptoms in the general population (12). In a comprehensive review of several population-based studies of the frequency of psychotic symptoms, it was found that over 8% of the population reports psychotic experiences and that these could be explained through the same etiological factors that also increase risk for non-affective psychoses (12).

In addition to the study of individual psychotic symptoms in the general population, Johns and van Os (15) proposed that there is a second approach to investigating population continua. That approach describes subclinical, attenuated trait-like expressions of the disorder ranging along a continuum in the form of schizotypal signs. This schizotypy approach is the focus of the present article^1^.

Together, the observation of individual psychotic symptoms and the description of subclinical schizotypal traits in the general population have led to the concept of the schizophrenia spectrum. This work is based on the notion of a non-binary distribution of schizophrenia (12), an assumption that is currently receiving widespread agreement in the literature (5).

Here, we examine the schizotypy approach in more detail and then focus on the overlap between schizotypy and schizophrenia. In doing so, we provide an overview of empirical work pointing to similarities between the two constructs not only at the level of phenomenology but also genetics, cognition, perception, motor control, and neurobiology. The argument proposed on the basis of these data is that schizotypal traits share not just superficial similarity with the signs and symptoms of schizophrenia but appear related to the clinical disorder at multiple levels of analysis.

## Phenomenology and Measurement of Schizotypy

The term schizotypy was coined by Rado (16). The large and methodologically heterogeneous literature on schizotypy can broadly be grouped into two approaches, viz. the so-called quasi-dimensional and fully dimensional approaches (6, 15, 17).

According to Meehl (18, 19), schizotypy is the psychological and personality organization of most, but not all, individuals with schizotaxia. Schizotaxia is defined by Meehl as a neural defect due to a synaptic aberration termed hypokrisia, which is caused by a single gene, the schizogene. According to Meehl, schizotaxia has a population base rate of about 10%. Meehl’s quasi-dimensional, psychiatrically oriented approach considers schizotypy as the subclinical expression of the symptoms of schizophrenia (20, 21). Evidence for this approach stems from taxometric studies (22), although there are also failures to provide evidence of a taxon (7).

The fully dimensional approach (23) is rooted in differential psychology and is based on the work of Eysenck and Claridge (7, 24–27). This approach treats schizotypy as a personality trait that is continually distributed in the general population. This trait shows individual differences and results, at its extreme high end, in a diagnosis of schizophrenia. Evidence for this approach stems from taxometric studies that consider positive skewness of sample distribution (7). The high prevalence of psychotic-like experiences in the general population is also in accordance with the fully dimensional approach (28, 29).

Theoretical issues aside, both approaches agree that there is variation in schizotypal features in the population, although specific details concerning the nature of the distribution (for example, the question of whether there is a taxon or not) have not been finally resolved (7, 15, 22).

Schizotypy can be assessed in the general population (30, 31) using clinical interviews [such as the Structured Interview for Schizotypy (32)] or psychometric self-report questionnaires [such as the Schizotypal Personality Questionnaire (SPQ (33)), the Oxford–Liverpool Inventory of Feelings and Experiences (O-LIFE (34)), the Rust Inventory of Schizotypal Cognitions (RISC (35)), the Community Assessment of Psychic Experiences (CAPE (36)), the Schizotypal Personality Scale (STA (37)), the Peters et al. Delusions Inventory (PDI (38)), the Eysenck Psychoticism (P) Scale (39), or the Chapman scales (40)]. Sample items from the SPQ are shown in Table 1.

**Table 1:** **Sample Questionnaire Items for the measurement of schizotypy**.

Item	SPQ dimension
Do you sometimes feel that things you see on the TV or read in the newspaper have a special meaning for you?	Ideas of reference (Pos)
I often hear a voice speaking my thoughts aloud	Unusual perceptual experiences (Pos)
I feel I have to be on my guard even with friends	Suspiciousness (Pos)
I sometimes jump quickly from one topic to another when speaking	Odd speech (Dis)
I sometimes use words in unusual ways	Odd speech (Dis)
My “non-verbal” communication (smiling and nodding during a conversation) is not very good	Constricted affect (Neg)
I prefer to keep myself to myself	No close friends (Neg)

*The items are taken from the Schizotypal Personality Questionnaire (SPQ). Pos: the positive dimension of schizotypy; Dis: the disorganization dimension of schizotypy; Neg: the negative dimension of schizotypy*.

The psychometric structure of schizotypy has been examined in numerous factor analyses of questionnaire data. Overall, there appears to be some agreement in the literature that the variance of schizotypy is well explained by a three-factor structure including the following dimensions (41). The “cognitive-perceptual” dimension (also referred to as positive schizotypy) includes perceptual alterations that bear some resemblance to the hallucinations reported in schizophrenia. It also includes unusual thoughts and views that resemble the delusions of schizophrenia. The “disorganized” dimension includes formal thought disorder and eccentric behavior. Finally, the “interpersonal” dimension (also referred to as negative schizotypy) is characterized by the loss of emotional, physical, and social functions such as pleasure, volition, interest in social contacts, and emotionality. Altogether the phenomenology of schizotypy thus possesses considerable similarity with the factor structure of schizophrenia (42).

The three-factor structure of schizotypy has been recovered independent of variables such as gender, culture, and religion (43). Individual differences in schizotypy can be measured with high reliability and possess high temporal stability (33–35, 44). It should also be noted that the scores of psychometric self-report questionnaires correlate highly with observer-ratings gained from clinical interviews (33, 45), further supporting the validity of these measures.

## Genetic, Cognitive, and Neurobiological Studies of Schizotypy: Overlap with Schizophrenia?

The description of schizotypy in the preceding section provides clear evidence of its phenomenological overlap with schizophrenia. This evidence is complemented by empirical studies further investigating such putative overlap on different levels of analysis, namely (1) genetic and non-genetic etiological influences, (2) cognition, perception, and motor control, (3) brain structure and function, and (4) psychopharmacology. In the following part of this article, we will provide an overview of that work. As will become clear from the following discussion, these studies provide strong support for similarities between schizotypy and schizophrenia across these multiple analysis domains.

Due to the large number of studies on schizotypy, the following review is not comprehensive. We do, however, provide a table summarizing the key details of all available functional magnetic resonance imaging (fMRI) studies of schizotypy (Table 2).

**Table 2:** **Summary of fMRI studies of psychometric schizotypy**.

Author	Field strength (T)	Task	Sample size	Groups	Schizotypy measure	Behavioral results	Neuronal results
Aichert et al. (46)	1.5	Antisaccade task	54	None	Rust Inventory of Schizotypal Cognitions	Significant positive correlation between schizotypy and antisaccade error rates	Significant negative correlation between schizotypy and activation in putamen, thalamus, cerebellum, and visual cortex (antisaccades) and visual cortex, supplementary eye fields, and posterior intraparietal sulcus (prosaccades)
Corlett and Fletcher (47)	3	Kamin blocking task	18	None	Chapman Scales and Peters et al. Delusions Inventory	No association between schizotypy and behavioral results	Significant negative correlation between schizotypy (Magical Ideation Scale) and striatal prediction error (PE) response; significant negative correlation between degree of distress with odd beliefs (PDI) and PE response in frontal cortex, striatum, and midbrain
Debbané et al. (48)	3	Self-reflection task	42	None	Schizotypal Personality Questionnaire	Females showed higher positive schizotypy scores than males. No association between schizotypy and behavioral results	Significant negative correlation between positive schizotypy scores and activation in left lingual gyrus in the self vs. other contrast. Significant positive correlation between positive schizotypy scores and activity in posterior cingulate cortex, dorsomedial prefrontal cortex, and dorsolateral prefrontal cortex in the self vs. control and other vs. control contrasts. Those associations were not due to sex differences
Ettinger et al. (49)	1.5	Sequence learning task	26	None	Eysenck Psychoticism Scale and Schizotypal Personality Scale	No significant correlations between EPQ-R/STA and procedural learning (PL) scores; when controlled for EPQ-R neuroticism, positive correlation between STA, and PL score	Significant positive correlation between psychoticism and activation in temporal cortex, striatum, thalamus, inferior frontal areas, middle frontal gyrus, and anterior cingulate; significant positive correlation between STA scores and activation in middle temporal gyrus.
Fink et al. (50)	3	Alternative uses task	41	21 high (range of SPQ scores: 132–179); 20 low (range of SPQ scores: 3–77)	Schizotypal Personality Questionnaire	No significant differences between low and high schizotypy in the originality of generated ideas. Generated ideas were correlated with self-report measure of ideation behavior and originality score of the Torrance Picture Completion Task.	High schizotypal individuals showed more activation than low schizotypal individuals during generation of alternative task (when compared to generation of common uses) in the left superior temporal gyrus and the right precuneus. Low schizotypal individuals showed more activation than low schizotypal individuals during generation of alternative task (when compared to generation of common uses) in the left/middle frontal, left inferior frontal and left inferior parietal regions and the anterior cingulate. Originality was associated with reduced deactivation of right parietal brain regions and the precuneus during creative ideation generation
Huang et al. (51)	3	Happy facial expression paradigm	28	14 high (SPQ mean = 45.79, SD = 1.84); 14 low (SPQ mean = 11.05, SD = 1.25)	Schizotypal Personality Questionnaire	No significant group differences.	High schizotypes showed significantly less deactivation to dynamic facial expressions that went from happy to neutral in anterior cingulate cortex and significantly higher deactivation in posterior cingulate cortex and superior temporal gyrus TG when faced with blame (vs. praise) cues than low schizotypes
Kumari et al. (52)	1.5	Prepulse inhibition (PPI)	14	None	Eysenck Psychoticism Scale	Significant negative correlation between psychoticism and PPI in 120 ms prepulse-to-pulse- interval, negative trend-level correlation for 30-ms PPI condition and for pulse-alone amplitudes	120-ms PPI condition: significant negative correlation between psychoticism scores and neural activation in inferior parietal lobe, insula, parahippocampal gyrus, inferior frontal and middle temporal gyri. 30-ms PPI condition: significant negative correlation between psychoticism scores and neural activation in inferior parietal lobe, insula, putamen, parahippocampal gyrus
Lagioia et al. (53)	3	Reality monitoring task	33	None	Schizotypal Personality Questionnaire	No association between schizotypy and behavioral results	Significant negative correlation between schizotypy and neural activation in left medial prefrontal cortex during retrieval of self/experimenter information (=origin information) compared to retrieval of contextual information
Lagioia et al. (54)	3	Resting state	39 (adolescents)	None	Schizotypal Personality Questionnaire	Not applicable	Positive correlation between SPQ measures and the visual network in the low frequency range (0.05 Hz) and negative correlation between SPQ measures and the auditory network in the high frequency range (0.16–0.19 Hz). Unlike in schizophrenia patients, the default-mode was unrelated to schizotypal trait expression
Modinos et al. (55)	3	Reappraisal task	34	17 high psychosis-prone (CAPE score above 75th percentile); 17 low psychosis-prone (CAPE score below 25th percentile)	Community Assessment of Psychic Experiences Questionnaire, positive subscale	Both groups reported successful diminishment of experienced negative emotion when instructed to reappraise a negative picture	High psychosis-prone subjects showed stronger activation in a number of prefrontal regions during reappraisal, relative to attending to negative pictures. The amygdala response to negative stimuli was decreased through reappraisal only in the low group. Functional connectivity analysis revealed less prefrontal-amygdala coupling in high psychosis-prone subjects.
Modinos et al. (56)	3	Self-reflection task	36	18 high psychosis-prone (CAPE score above 75th percentile); 18 low psychosis-prone (CAPE score below 25th percentile)	Community Assessment of Psychic Experiences Questionnaire, positive subscale	High psychosis-prone subjects attributed less positive traits to others than subjects with low psychosis-proneness	Across subjects there was more activation in cortical midline structures and insula in the self-semantic compared to the self-other condition. This was not the case for the other-semantic condition. There was more activation in the posterior cingulate cortex in the other compared to the self-induced condition in low psychosis-prone subjects but not high psychosis-prone subjects. High psychosis-prone subjects showed increased activation in left insula, right dorsomedial prefrontal cortex, and left ventromedial prefrontal cortex for positive self-related traits. They showed increase in activation in bilateral insula, anterior cingulate and right dorsomedial prefrontal cortex for negative self-related traits
Modinos et al. (57)	3	Theory of mind task	36	18 high psychosis-prone (CAPE score above 75th percentile); 18 low psychosis-prone (CAPE score below 25th percentile)	Community Assessment of Psychic Experiences Questionnaire, positive subscale	No between-group differences were found on behavioral performance.	When compared to low psychosis-prone subjects, high psychosis-prone subjects showed more activation in the anterior prefrontal cortex (BA 10) during second order mentalizing than in first order mentalizing. When compared to low psychosis-prone subjects, high psychosis-prone subjects showed more activation in the dorsomedial and lateral prefrontal regions during second order mentalizing than in first order mentalizing
Mohanty et al. (58)	1.5	Emotional Stroop task	32	16 high positive schizotypals (>1.5 SD above mean on Perceptual Aberration Scale or Magical Ideation Scale); 16 low positive schizotypals (<0.5 SD above mean on Perceptual Aberration Scale or Magical Ideation Scale)	Chapman Perceptual Aberration Scale; Chapman Magical Ideation Scale	No significant group differences	In comparison to low positive schizotypals, high positive schizotypals showed increased right and decreased left activity in dorsolateral prefrontal cortex in response to negative as opposed to neutral condition. They also showed abnormal activity in ventral limbic areas, including decreased activity in nucleus accumbens and increased activity in hippocampus and amygdala in negative as opposed to neutral condition
Premkumar et al. (59)	1.5	Social rejection task	26	12 with high score (≥7) on Unusual Experiences subscale of O-LIFE; 14 with low score (≤2) on Unusual Experiences subscale of O-LIFE	Oxford Liverpool Questionnaire of Feelings and Experiences	Subjects with high unusual experiences score had higher scores in cognitive disorganization and impulsive non-conformity. They also reported more negative current mood and higher anxiety and rejection sensitivity	Across all subjects a temporo-occipito-parieto-cerebellar network was active during rejection and a left fronto-parietal network during acceptance, relative to neutral scenes. The bilateral lingual gyrus was more active during rejection relative to acceptance scenes. In subjects with low unusual experiences scores dorsal anterior cingulate and dorsoventral prefrontal cortex was activated in response to social rejection stimuli. Subjects with high unusual experiences scores showed deactivation in dorsal anterior cingulate and dorsoventral prefrontal cortex in response to social rejection stimuli

*The table shows, in alphabetical order by first author, all fMRI studies of schizotypy. Field strength is given in Tesla (T). SPQ = Schizotypal Personality Questionnaire. O-LIFE: Oxford Liverpool Questionnaire of Feelings and Experiences. CAPE: Community Assessment of Psychic Experiences. EPQ-R: Eysenck Personality Questionnaire – Revised. STA: Schizotypal Personality Scale. PPI: prepulse inhibition*.

### Genetic and environmental risk factors

Behavioral genetic studies estimate the heritability of schizophrenia to be approximately 66–83% (60) and that of schizotypy to be around 30–50% (61–64), although lower estimates have also been reported for schizotypy (65). Evidence of genetic overlap between schizophrenia and schizotypy comes from family studies, which show that first-degree relatives of schizophrenia patients have increased levels of schizotypy (66, 67), particularly in the negative dimension (68). In addition to increased mean levels of schizotypy in the relatives compared to controls without a first-degree relative with schizophrenia, there are also reports of associations between the profile and severity of clinical symptoms in the patients and the dimensions of schizotypy in the relatives (69). For example, one study showed that the severity of positive symptoms in psychotic patients was associated with the level of positive schizotypy in the relatives, similar to the findings of another study on negative schizotypy (70). Overall, these studies provide evidence for genetic, or at least familial, correlations between schizotypy and schizophrenia.

More recent molecular genetic data complement these behavioral genetic studies. For example, a genome-wide association study (GWAS) showed considerable overlap between the genetic association profiles of schizophrenia and schizotypy (71). Put together, these molecular genetic studies point to considerable overlap of schizotypy with schizophrenia at the genetic level by implicating similar polymorphisms in both phenotypes.

In addition to genetics, environmental risk factors are also known to play an important role in the etiology of schizophrenia, including winter or spring birth (72), growing up in an urban area (73), being an ethnic minority (74), childhood trauma (75), and cannabis use (76). These factors also increase the risk for subclinical spectrum phenotypes, again supporting the notion of overlap in etiological factors between schizophrenia and its subclinical expression (15).

### Cognition, perception, and motor control

A large body of work has documented cross-domain deficits in automatic and controlled cognition, perception, and motor control in schizophrenia. Meta-analyses suggest that the most pronounced deficits are in the domains of verbal learning and memory, working memory, attention and executive functions as well as psychomotor control (77).

Complementing the work on schizophrenia, there have been numerous investigations of cognition, perception, and motor control in relation to schizotypy. The overall finding that emerges from these investigations is that high levels of schizotypy are associated with subtle performance deficits, mostly compatible with the pattern of findings observed, albeit at greater severity, in schizophrenia. The literature on cognition has been qualitatively reviewed in a number of recent publications (6, 17, 21). A comprehensive meta-analysis of the association between all aspects of cognition, perception, and motor control with schizotypy using different study designs is missing, although a recent study calculated effect sizes for measures of cognition in a selection of studies that included a psychometrically defined schizotypy group drawn from the population of college students (78). That analysis did not find a strong effect size for the comparison between schizotypy groups and controls, but is limited not only by the sample inclusion criterion but also the exclusion of neurocognitive measures such as prepulse inhibition and oculomotor tasks, measures known to be reliably associated with schizotypy (see below).

Here, the key findings of studies examining the association between schizotypy and cognition, perception, and motor control will be highlighted briefly. These studies have generally followed one of two designs, by either comparing groups of individuals with high scores on psychometric schizotypy questionnaires to those with medium or low scores on these questionnaires in an extreme groups approach or by investigating correlations between schizotypy questionnaire scores and task performance in samples not selected *a priori* according to specific schizotypy cut-offs.

Regarding cognition, these studies have shown that higher levels of schizotypy are associated with impairments in working memory (79–84), in executive functions [Wisconsin Card Sorting Test, WCST: (79, 85–87); verbal fluency: (88)], in early sensorimotor filtering in the prepulse inhibition paradigm [PPI; (52, 89–91)], in visual backward masking (87), in the flexible adaptation of behavioral control following cognitive conflict (92), in the latent inhibition learning paradigm (93), in the recognition and naming of emotional facial expressions (94, 95), in attention [Continuous Performance Test, CPT; (96, 97)], in perspective taking regarding visual stimuli (98) as well as one’s own body (99, 100), and in Theory of Mind (101). The deficits observed on these tasks are generally similar in kind to those observed in schizophrenia.

In addition, schizotypy is also associated with perceptual deficits, such as in the discrimination of auditory (102) and olfactory stimuli (103), and motor impairments. Motor impairments similar to those observed in schizophrenia were found by assessment of neurological soft signs (104, 105), gait (106), and the precision of manual motor control (107). Moreover, the smooth pursuit and antisaccade eye movement deficits that are well described in schizophrenia (108, 109) are also observed in schizotypy [antisaccades: (110–112); smooth pursuit (112–115)]. These include a reduced ability to match eye velocity to target velocity in the smooth pursuit task and the failure to inhibit automatic saccades to a peripheral target in the antisaccade task.

The levels of impairment in different cognitive domains have also been related to the participants’ specific schizotypy profile. For example, it was found that people with negative schizotypy (88, 116–118) as well as disorganized features (87, 88) are particularly impaired in frontally mediated executive functions, whereas cognition and behavior of people with positive schizotypy suggest impairments in temporo-limbic circuits (117). Gait abnormalities were also observed in positive but not negative schizotypy (106).

Of importance in this context is the observation that at least some of these neurocognitive deficits survive statistical correction for factors such as intelligence (88, 92) and neuroticism (92, 110), providing further support for the existence of genuine cognitive impairments in people with high levels of schizotypy, over and above these measures of general (cognitive or emotional) functioning.

In contrast to the deficits described above, there are only relatively few cognitive functions that are not found to be impaired in schizotypy. For example, relatively inconsistent findings are observed in the negative priming paradigm (89, 119) and the Stroop task (89, 120–126). Additionally, Raine (41) argues that the level of general intelligence is relatively unimpaired in schizotypy. Finally, there are reports of cognitive domains where higher levels of schizotypy are associated with better performance, for example, in measures of creativity (127–129).

### Brain structure

Since the early observation of enlarged ventricles (130) in schizophrenia, there have been numerous computed tomography (CT) and structural magnetic resonance imaging (MRI) studies of the brain in this disorder. A number of reviews of this literature are available (131, 132). Overall, these reviews have shown that patients with schizophrenia, when compared to healthy controls, show gray matter volume reductions in a number of brain areas. These volume reductions are most pronounced in temporal, parietal, and frontal cortical areas, hippocampus, amygdala, and cerebellum. Additionally, there is evidence of ventricular enlargement. Taken together, these results point to a non-localized pattern of structural changes, compatible with the hypothesis of schizophrenia as dysconnectivity syndrome (133–135).

Extending this line of research into schizotypy, structural MRI studies have investigated associations between the level of schizotypy and measures of gray and white matter volume in clinically unaffected individuals with the aim to search for schizophrenia-like volumetric changes in high schizotypy. An early 0.15 T MRI study of 17 healthy subjects showed that higher overall levels of several schizotypy scales were associated with a lower area of the left prefrontal cortex as well as a lower area ratio of prefrontal cortex to temporal cortex (86), compatible with prefrontal volume reductions in schizophrenia. A more recent study, however, showed that healthy individuals with higher levels of positive schizotypy (measured using the CAPE) had larger whole-brain volumes as well as posterior cingulate and precuneus volumes (136). This positive association, which is not in agreement with the hypothesis of similar brain structural deficits in schizotypy and schizophrenia, possibly suggests that protective or compensatory mechanisms are operational in people with high levels of schizotypy but without the diagnosis of schizophrenia. Such a conclusion is in agreement with another structural MRI study, which reported a positive association of overall schizotypy levels (SPQ) and cortical thickness in the frontal lobe (137). However, the same study also observed a negative association between schizotypy and thalamic volume (137), compatible with reports of thalamic volume reductions in schizophrenia (138, 139). Finally, we recently observed that higher levels of positive schizotypy (35) were associated with volume reductions in frontal and temporal cortical areas, significantly overlapping with the pattern of volumetric reductions typically observed in schizophrenia (140).

These investigations of gray matter volumes in schizotypy have been most recently complemented by MRI studies of white matter. A diffusion tensor imaging (DTI) study (141) compared people with high MMPI-2 (Minnesota Multiphasic Personality Inventory) mean scores in the psychosis-like Sc (Schizophrenia), Pa (Paranoia), and Pd (Psychopathic Deviate) scales with controls with low scores. The group with high scores had higher fractional anisotropy (FA) in the left arcuate fasciculus, but lower FA in the right arcuate fasciculus in fronto-parieto-temporal tracts and in the corpus callosum. Another study (142) showed that higher cognitive–perceptual factor scores of the SPQ were associated with lower white matter integrity in fronto-temporal tracts, in the bilateral anterior thalamic radiation and the forceps minor.

In addition to these studies of psychometric schizotypy, there are a number of MRI studies of patients with a clinical diagnosis of schizotypal personality disorder (SPD^2^), a clinical disorder with a population prevalence of approximately 3.9%. The findings from these studies have recently been reviewed in detail elsewhere (143, 144) and are only summarized briefly here. In short, volume reductions in SPD are seen in the temporal lobe, similar to findings from schizophrenia. However, it has also been found that SPD patients do not display the frontal lobe volume reductions observed in schizophrenia, possibly reflecting protective or compensatory processes. Detailed investigations of subcortical structures support this claim: both schizophrenia and SPD show volume reductions in the thalamic pulvinar, a structure that shows prominent connections with the temporal lobe, but not in the mediodorsal nucleus, which is primarily connected with the frontal lobe (145). A recent study yielded distributed gray matter volume reductions in frontal, parietal, and temporal areas in an important sample of antipsychotic-naïve men with SPD (143), suggesting that these deficits are not due to (long-term) antipsychotic treatment.

### Brain function

A still relatively small – but growing – body of work has used fMRI to investigate the functional neuronal correlates of schizotypy. The key features and findings from these studies are summarized in Table 2.

Amongst these studies, there is concrete evidence of similarity in neural activation patterns between schizophrenia and schizotypy. For example, the above mentioned PPI paradigm, a measure of early attentional processing, was found to be associated with reduced activation in the insula, putamen, thalamus inferior parietal cortex, hippocampal gyrus, and fusiform gyrus in persons with high levels of psychoticism (P) (52). These activation reductions resemble the data obtained from samples of patients with schizophrenia (146, 147).

Further evidence for brain functional similarity between schizotypy and schizophrenia was reported by Corlett and Fletcher (47). In an fMRI study of the Kamin blocking task, these authors observed an association of lower BOLD signal in the striatum during unexpected “prediction error” trials with higher schizotypy scores and an association between distress with unusual beliefs and variation in prediction error related signal in the right frontal cortex. The latter finding is directly compatible with data from earlier investigations of the prediction error effect in schizophrenia (148) and following administration of ketamine (149), a non-competitive NMDA receptor antagonist known to cause psychosis-like states (150).

Additionally, the already mentioned behavioral deficits in antisaccade performance in schizotypy were found to be associated with reduced activation levels in the striatum, thalamus, cerebellum, and occipital cortex (46). This pattern resembles fMRI studies of volitional saccades in schizophrenia patients [striatum: (151–153)] and their unaffected relatives [striatum: (154), occipital cortex: (155)], although reductions in frontal lobe activation levels were additionally described in schizophrenia (152) but not in schizotypy.

Finally, qualitative alterations in the neural processing patterns underlying a number of other paradigms have also been found in schizotypy, e.g., in an emotional Stroop task in students with high levels of positive schizotypy (58). A series of publications on a sample of students with high CAPE scores showed (i) increased activation in prefrontal areas in a second order Theory of Mind task despite unimpaired behavioral performance (55), (ii) reduced prefrontal control of emotional processing (57), and (iii) altered activation patterns during the processing of self-related stimuli (56). In another study, people with high levels of schizotypy were found to deactivate the dorsal anterior cingulate cortex (ACC) whilst viewing pictures of social rejection, similar to what has been found in depressed patients, whereas controls activated the same structure (59). Huang and colleagues (51) on the other hand found that people with high SPQ scores showed weaker deactivations in right ACC and stronger deactivations in left posterior cingulate cortex and right superior temporal gyrus (STG) when viewing happy faces. Debbané and colleagues (48) examined the neural correlates of self-reflective processes and positive psychometric schizotypy in adolescence using a trait evaluation task. A negative association was found between schizotypy and activation in left lingual gyrus while the participants were rating themselves compared to others. During the ratings of self vs. control and other vs. control, schizotypal traits were positively correlated with activity in posterior cingulate cortex and dorsolateral and dorsomedial prefrontal cortex. Using a reality monitoring task in adolescents an association was found between higher SPQ scores and reduced activation in BA10 during judgments of whether a word was generated by the participant or the experimenter (53). In a study on the association between high positive schizotypy and creativity, Fink and colleagues (50) found similarities in the brain activation between schizotypy and creativity (as measured by originality) during creative ideation. Both high scores on the O-LIFE unusual experiences scale and originality were associated with reduced deactivation in right parietal brain regions and the precuneus during creative idea generation. Finally, the implementation of a dopamine-sensitive procedural learning task in fMRI yielded positive associations between EPQ-R (39) psychoticism scores and BOLD signal in striatum, thalamus, insula, and frontal cortex as well as between STA scores (37) and BOLD signal in right temporal cortex (49).

The observation that stress is a possible risk factor for schizophrenia (156) has led to an fMRI study of the neuronal response to stress in participants with high schizotypy (157). Differences in striatal and limbic activation patterns were found in participants with negative schizotypy in comparison to controls. These findings complement an earlier positron emission tomography (PET) study by the same group, in which participants with negative schizotypy were found to show greater stress-induced striatal dopamine release (158).

Apart from these studies of cognitive or emotional activation paradigms, more recently the investigation of brain function at “rest,” i.e., in the absence of an overt task, has become increasingly instructive. Patients with schizophrenia have been found to show alterations in the resting-state activation patterns (159). The only resting-state fMRI study of schizotypy reported positive correlations between SPQ scores and a visual network in the low frequency range as well as negative correlations between SPQ scores and an auditory network in young people aged 12–20 years (54).

Several fMRI studies of SPD are also available. A first study by Koenigsberg and colleagues (160) showed that patients with SPD displayed reduced activations in distributed fronto-parietal areas during the retention interval of a visuo-spatial working memory task. Dickey et al. (161) found that auditory discrimination, a measure also known to be impaired in schizophrenia, was associated in SPD with hyper responsive neuronal processing of deviant tones in temporal and parietal areas. In another study on auditory processing Dickey et al. showed (162) that the recognition of emotions (sad, happy, sarcastic, neutral) in the prosody of semantically neutral sentences was unimpaired in SPD; however, processing was associated with seemingly inefficient activation in the STG.

The relationship between schizotypy and brain function has also been investigated using electroencephalography (EEG). For example, it was found that high SPQ schizotypy is associated with a reduction in the P100 amplitude during a working memory task, suggesting the existence of early information processing deficits (81), similar to what had previously been found in schizophrenia (163). However, EEG studies have also pointed to later processing deficits. Klein and colleagues (164) observed a reduction in P300b amplitude in participants with higher overall schizotypy (SPQ), complementing P300 amplitude changes that had been reported in patients with schizophrenia and their relatives (165). In another study, stronger negativity in the N400 window was found to be associated with higher scores in the SPQ disorganized dimension (166). Koychev and colleagues (167) studied a working memory task and found that participants with high SPQ schizotypy had reduced network synchronization in the beta and gamma range in fronto-central and central-occipital regions, compatible with the observation of disturbed cortico-cortical connectivity in schizophrenia. Another study (99) found that the behavioral deficit in bodily perspective taking in schizotypy (168) was associated with an increased duration of activity of the right temporo-parietal junction. Additionally, there is a replicated association between reduced P50 suppression in EEG and increased schizotypy (169, 170), especially in the disorganized dimension (171), complementing findings of reduced P50 suppression in schizophrenia patients, their relatives, and SPD patients (172, 173).

Finally, a number of studies have employed functional near-infrared spectroscopy (fNIRS) to probe relationships between brain function and schizotypy. These studies showed that (i) increased levels of divergent thinking in schizotypy is associate with stronger activity in right prefrontal cortex in healthy and schizophrenic subjects (129), (ii) higher overall schizotypy scores (SPQ) are associated with reduced activity in frontal cortex during the recognition of one’s own face but with increased activity during the “Reading the Mind in the Eyes” task (174), and (iii) high schizotypal (SPQ) participants show stronger activity and a greater right > left asymmetry during a letter fluency task (175, 176).

### Psychopharmacology

Psychopharmacological methods have also been drawn upon to elucidate the neural basis of schizotypy as well as possible similarities with schizophrenia. A central observation in schizophrenia research is the finding of increased striatal dopamine release that is seen in the patients following the acute administration of amphetamine. Imaging studies have shown that patients with schizophrenia, both in the acute phase and in remission, show amphetamine-induced reductions in D2 and D3 dopamine receptor binding potential in the striatum (177, 178). Similar findings were obtained in patients with schizotypal personality disorder (179). Importantly, a relationship between striatal dopamine release after amphetamine and SPQ schizotypy was observed in a sample of non-clinical volunteers (180), providing support for a shared dopaminergic dysfunction in schizotypy and schizophrenia.

In a large, multi-center pharmacological study of SPQ schizotypy it was observed that the D2/D3 receptor antagonist amisulpride improved working memory and verbal fluency performance in persons with high levels of schizotypy but worsened it in medium schizotypal controls (181). Data from the same study showed that administration of 7 mg risperidone led to deterioration in antisaccade task performance in medium schizotypy controls, whereas in high schizotypy a non-significant tendency toward improvement was observed (182), compatible with the improvements in antisaccade performance seen in schizophrenia with risperidone treatment (183, 184). Together, these data suggest that persons with high schizotypy benefit from antipsychotic compounds similar to patients with schizophrenia, or at least tolerate them, whereas controls do not. Finally, an EEG study of the semantic processing of words (185) showed that anterior components of the increased N400 potential in high schizotypy were reduced through a one-off administration of the antipsychotic olanzapine. This effect resembles findings from schizophrenia research (186, 187) and suggests a dampening of increased salience in high schizotypy, as in low schizotypal subjects there was no such effect of olanzapine.

Taken together, these pharmacological studies yield highly interesting evidence of similarity between schizotypy and schizophrenia with respect to the neurocognitive effects of psychotomimetic and antipsychotic compounds. However, evidence of differences between these groups has also been obtained. For example, the dopamine increasing substance levodopa leads to improvements in a mental rotation task in people with high positive schizotypy, whereas cognitive improvements in schizophrenia tend to be observed after treatment with dopamine antagonists. This pattern of effects was explained by drawing upon the non-linear relationship between dopamine and performance along an inverted U-function (106).

### Summary of genetics, cognition, and neurobiology

Overall, the studies described in the previous sections provide support, across a number of methods and functional domains, of alterations in cognition, perception, motor control, and neurobiology in schizotypy. Importantly, many of these alterations are qualitatively similar to the impairments seen in schizophrenia, supporting the notion of similarity between schizotypy and schizophrenia. However, evidence of discontinuities is also reported in some cognitive tasks that do not consistently show impairments in schizotypy, such as the negative priming and Stroop tasks. Additionally, better performance in schizotypy has been observed on measures of creativity and some structural neuroimaging evidence exists of possibly protective or compensatory neural resources in schizotypy. Clearly, these putatively preserved (or enhanced) functions and structures need to be investigated further.

## Schizotypy: Importance and Implications for Schizophrenia Research

Taken together, the cited studies support the assumption of a continuum between schizophrenia and schizotypy at different levels of analysis (6). Accordingly, implications of our current understanding of schizotypy to schizophrenia research are based at least in part on the continuum hypothesis, as detailed in the following section.

First, the documentation of such a continuum provides important clues regarding the etiology of schizophrenia (5, 15). Continuously distributed (and reliably measured) phenotypes are suggested to be the result of multiple etiological factors. A multifactorial etiology of schizophrenia has frequently been postulated. Accordingly, current research focuses on multiple gene loci (188) as well as several environmental risk factors (156). Of further interest in this context is the assumption that highly schizotypal individuals may be undetected carriers of schizophrenia risk alleles, which may provide one answer to questions from evolutionary biology concerning the frequency of schizophrenia in the population (128, 189).

Second, in addition to providing clues regarding the etiology of schizophrenia, schizotypy is an important topic for investigations in its own right, not only because of the documented neurocognitive impairments but also because of its associations with a number of maladaptive behaviors and psychiatric symptoms. For example, high levels of schizotypy are associated with cigarette smoking (190–192) and consumption of drugs such as cannabis (193, 194), negative affect, post-traumatic stress disorder, anxiety disorders and depression (195–198), and lower social, educational, and professional levels of functioning (197, 199–207). Overall, it can be concluded that people with high levels of schizotypy display maladaptive behaviors and psychiatric symptoms and suffer a lower quality of life (197, 208). Therefore, a thorough characterization of the cognitive and neural correlates of schizotypy can lead to an improved understanding of these disturbances and development of appropriate therapies.

Third, there may also be merits of studying schizotypy as a model system of schizophrenia. A particularly promising application of such a model system is in relation to antipsychotic and pro-cognitive drug development, an area of clear need in the treatment of schizophrenia (209, 210). The methodological advantages of such a model system include the availability of cheap, reliable, and objective psychometric questionnaires (17, 190). Additionally, continuously distributed data possess high statistical power (211). Finally, studies of high schizotypes are not confounded by problems such as long-term pharmacological treatment or chronic hospitalization as is frequently encountered in schizophrenia research.

Fourth, some authors have argued that a conceptualization of schizophrenia as a spectrum disorder may lead to increase in psychoeducational and psychotherapeutic treatment success. For example, David (5) and Johns and van Os (15) argue that dimensional definitions of the symptoms of schizophrenia are less stigmatizing than categorical diagnoses, a factor that could contribute beneficially to treatment outcome.

Overall it has been argued (190, 212) that the continuum approach to etiological research possesses higher validity than the categorical approach. The latter, on the other hand, may yield better reliability and has of course unrivaled usefulness in clinical communication and decision making with regards to treatment strategies.

It is also important to point out differences between the schizotypy paradigm and other phenotypic and genotypic spectrum sample strategies within the wider context of schizophrenia research. For example, the popular and successful approach of studying the biological relatives of schizophrenia patients draws upon the principle of a genetic continuum (213). Two fundamental findings stemming from this large body of work are (i) the observation of schizophrenia-like signs and symptoms in the relatives and (ii) the identification of neural and cognitive endophenotypes. Endophenotypes, or intermediate phenotypes, are markers of the genetic vulnerability to the illness and may help to improve our understanding of the neuronal and behavioral effects of risk genes (214, 215).

A second, important schizophrenia-spectrum population that is currently under intense investigation is individuals at particularly high risk for developing the illness. Such individuals are identified from the general population on the basis of different trait and state factors and have been found to have a high frequency of conversion to schizophrenia (216), higher than that observed in psychometric schizotypy (217).

The schizotypy approach described here complements these genetic and high-risk approaches by adopting a psychometric criterion for the definition of this spectrum population.

## Conclusion

In this article, we reviewed evidence of putative overlap between schizotypy and schizophrenia. We argued first that this overlap is apparent at the phenomenological level, with schizotypy traits resembling, in attenuated form, the signs and symptoms of schizophrenia. Importantly, we then provided evidence from genetic, cognitive, and neurobiological studies supporting the assumption that schizotypy shows significant similarity with schizophrenia also at these levels, suggesting that the observed similarity at the phenomenological level may not be trivial. Future work will be required to further characterize the boundary between high schizotypy and schizophrenia. Another area of research that deserves further attention concerns the identification of differences between schizotypy and schizophrenia. These may point to protective or even compensatory mechanisms that prevent high schizotypes from developing full-blown schizophrenia.

## Conflict of Interest Statement

The authors declare that the research was conducted in the absence of any commercial or financial relationships that could be construed as a potential conflict of interest.

## Footnotes

^1^In this context, it is also important to point out that there are associations between the existence of individual psychotic symptoms and schizotypal traits (44, 218, 219), which speak for the integration of these two literatures. Such observations are to be expected under the assumption that higher trait schizotypy scores along the continuum from normal personality to schizophrenia will be associated with increasing manifestations of psychosis.

^2^It should be pointed out here that high levels of schizotypy on psychometric self-report questionnaires may result in a diagnosis of SPD, but not necessarily so. For example, Raine (33) reported that a diagnosis of SPD was made amongst 55% of people with SPQ scores in the top 10% of the distribution (compared to 0% of people from the lowest 10% of the distribution). According to ICD-10, a personality disorder is characterized by an inflexible, rigid personality that leads to considerable psychological strain. Therefore, it is important to mention that although all patients with a SPD diagnosis are likely to have high levels of schizotypy on a psychometric questionnaire, not all high psychometric schizotypes meet the diagnostic criteria for SPD.
